# Halothane-induced hepatitis: A forgotten issue in developing countries: Halothane-induced hepatitis

**Published:** 2011-01-01

**Authors:** Peiman Habibollahi, Nastaran Mahboobi, Sara Esmaeili, Saeid Safari, Ali Dabbagh, Seyed Moayed Alavian

**Affiliations:** 1 Department of Gastroenterology, Tehran University of Medical Sciences, Tehran, IR Iran; 2 School of Medicine, School of Public Health, Tehran university of Medical sciences, Tehran, IR Iran; 3 Department of Anesthesiology, Tehran University of Medical sciences, Tehran, IR Iran; 4 Anesthesiology Research Center, Shahid Beheshti University of Medical sciences, Tehran, IR Iran; 5 Baqiyatallah Research Center for Gastroenterology and Liver Diseases, Baqiyatallah University of Medical Sciences and Tehran Hepatitis Center, Tehran, IR Iran

**Keywords:** Halothane, Hepatitis, Halogenated, Anesthetics

## Abstract

Halothane was introduced as an anesthetic in the 1950s and was considered a revolutionary agent in the field of anesthesia. Soon after, halothane-induced hepatitis became a concern, leading to the development of less toxic gases that induced a lower incidence of side effects. Two types of halothane-related hepatotoxicity have been described: type 1, or mild hepatitis, is associated with elevated transaminase levels and self-limiting symptoms, and type 2, or severe hepatotoxicity, is associated with acute fatal liver failure and is fatal in most cases. Hepatotoxicity is most likely to be immune-related, based on much evidence. Free radicals that are produced by the metabolism of halothane in the liver can modify cellular proteins and introduce neo-antigens to the immune system. Sensitization to these neo-antigens induces a more severe response after multiple exposures; most cases of type 2 hepatitis occur after repeated contact. New halogenated anesthetics such as enflurane, sevoflurane, and desflurane, are not metabolized in the liver, causing few cases of sensitization. Compared with halothane, these anesthetics are expensive. As a result, replacement of halothane with new halogenated anesthetics requires a precise cost-benefit analysis, especially in developing countries that have low health care budgets.

## Background

Halothane was synthesized by the British chemist Charles Suckling over 2 years of research and testing. Its required concentration for anesthesia was predicted, and the drug was tested by a well-known anesthesiologist, Michael Johnston, who recognized its superior properties compared with other anesthetics that were available at that time [1]. Development of halothane was considered as a great progress in anesthesiology, and it was introduced globally in 1956 [2]. Subsequently, several reports of postsurgical lethal hepatotoxicity in patients who were anesthetized with halothane emerged [3][4][5][6]. Two types of halothane hepatotoxicity have been described. Type 1, or mild hepatitis, is associated with elevated transaminase levels and self-limiting symptoms, and type 2, or severe hepatotoxicity, is associated with acute fatal liver failure, resulting in death in most cases. In addition, seeking an ideal drug that was associated with pleasant induction and rapid recovery, these reports led to the synthesis of new agents with greater efficacy and safety. Methoxyflurane was introduced in the 1960s and was popular for several years, but its dose-related nephrotoxicity prompted the development of newer agents [7][8][9]. Several members of this family, such as enflurane [10], isoflurane [11], desflurane [12] and sevoflurane [13] were introduced sequentially. Despite the synthesis of new agents with a lower incidence of hepatitis-related side effects and development of techniques for faster induction and rapid recovery [14], their high cost has limited their use, especially in developing countries. Yet, the actual incidence of halothane-induced hepatitis in large-scale studies has not been significant [15]. Hepatitis has also been reported with newer agents, such as desflurane, sevoflurane, and isoflurane [16][17][18]. Thus,  substitution of halothane with newer agents and its financial benefits must be examined, especially in developing countries [19]. We performed a literature search in Pubmed, Medline, and Scopus using the following keywords and their combinations: halothane, hepatitis, inhalational anesthetics, and halogenated anesthetics. Relevant data, focusing on halothane-induced hepatitis and comparing halothane with new halogenated anesthetics were extracted and reviewed.

## Mechanism

The exact mechanism of halothane-induced hepatotoxicity is unknown, but there is strong evidence that the immune system mediates hepatitis-related mortality [20]. Increased levels of circulating immune complexes, rash, and arthralgia, which implicate involvement of the immune system, are associated with halothane-induced hepatitis [21][22]. In the serum of affected patients, antibodies against hepatocytes exist [23][24]. Based on these observations, it has been hypothesized that the immune system mediates the development of hepatotoxicity. On the other hand, greater severe liver dysfunction and necrosis is reported among patients with repeated exposure to halothane [25], which has strengthened this hypothesis. Known human risk factors such as female gender, adulthood, and genetics have been used to develop an animal model of halothane hepatitis, in which severe hepatitis with lesions, similar to halothane hepatitis in humans, has been produced [26]. Although more severe form of hepatic damage is believed to be immune-mediated, mild forms of hepatic injury following halothane exposure are attributed to the intracellular metabolism of halothane and local alterations in hepatic blood flow and oxygen demand, resulting in hepatic hypoxia [27]. Halothane undergoes hepatic oxidative or reductive metabolism to a greater extent than other inhalational anesthetics [27][28], which is primarily mediated through cytochrome P450 (CYP450) [29][30][31]. Under normal conditions, up to one-third of halothane is metabolized through the oxidative pathway and catalyzed by cytochrome P450 2E1 and 2A6 while;less than 1% is metabolized through the reductive pathway [27][30][32].Trifluoroacetylchloride (TFA), an extremely reactive molecule, is generated by the reaction between lysine and halothane metabolites [30][31][32], which yields neo-antigens by binding to the hepatocyte macromolecules [33][34][35][36]. These neo-antigens can provoke immune responses against hepatocytes and induce fulminant hepatitis [22][29][37]. Neo-antigens can also be expressed on Kupffer cells, rendering the antigen-presenting cells [38][39]. These results implicate the immune response in the development of fulminant halothane-related postsurgical hepatic dysfunction. Lethal hepatocyte necrosis rises in serum aminotransferase levels, lipid peroxidation, and inactivation of CYP450 can be induced by free radicals that are produced during nonoxidative halothane metabolization [40][41][42]. This can be considered for milder forms of halothane-induced hepatitis, because only a small amount of halothane is degraded through non-oxidative pathway [43][44]. Although less frequent, hepatic failure has been reported following exposure to enflurane [45], isoflurane [46] and desflurane [47]. Only 2.4% of enflurane is degraded [48], causing few cases of hepatotoxicity [49][50]. Less than 0.2% of desflurane is metabolized [51]. In contrast, desflurane is strongly stable, with just 0.01% metabolism [52]. Consequently, based on the model of immune-mediated hepatotoxicity, Desflurane should be the ideal inhaled anesthetic, because it causes the least free radical formation.

## Manifestation

Two types of halothane-induced hepatotoxicity have been described, based on the severity of symptoms: mild hepatitis with elevations in aminotransferase levels and self-limiting symptoms [53][54][55], and severe hepatitis (or acute liver failure). Halothane degradation products and local hypoxia, due to insufficient blood supply and altered oxygen consumption, are chief causes of mild form of hepatotoxicity [27]. This condition usually presents as elevated liver transaminase levels following surgery. Some patients develop self-limited rash, arthralgia, lethargy, nausea and fever [15][56][57][58]. common manifestations of sever halothane-induced hepatitis are fever, anorexia, nausea, myalgia, arthralgia and rash Common manifestations of severe halothane-induced hepatitis [59]. Hepatomegaly and jaundice are also frequently observed, and eosinophilia has been reported in approximately 40% of patients, highlighting the immunological basis of this condition [60].

## Diagnosis

Halothane-induced hepatitis is a diagnosis of exclusion. Elevated serum aminotransferase levels, rash, arthralgia, jaundice and hepatomegaly are observed in various hepatic diseases and do not necessarily confirm a diagnosis. In contrast, other possible diagnoses such as surgical complications, preoperative hypotension, sepsis, infection, hepatotoxicity due to other drugs and viral hepatitis, should be considered.

## Treatment

Most cases of halothane-induced hepatitis are managed by supportive care [54]. Most mild cases do not need intensive care and resolve spontaneously with little intervention, but more severe fulminant hepatitis requires intensive care. Several treatment modalities such as urgent liver transplantation are efficacious in these patients [54]. Resolution of jaundice has been reported following methionine therapy in several case reports whereas no well-designed studies on this technique have been performed [61]. Corticosteroids do not have proven efficacy, and they might exacerbate the course of hepatitis [61]. Patients are typically managed according to the complications of acute liver failure, such as hepatic encephalopathy, cerebral edema, renal failure and metabolic disturbances.

## Prevalence

The estimated incidence of halothane-induced hepatitis varies between studies. The largest study was conducted by the National Institute of Health, which reviewed approximately 250,000 cases of halothane exposure; the incidence of severe life-threatening hepatic failure was 1/35,000 [25]. The incidence rate has been reported to range between 1/6000 and 1/35,000 [62]. One of the most significant risk factors of severe hepatitis is re-exposure to it. After several exposures, these rates can reach 1/1400 [25]. A genetic predisposition has been demonstrated in patients with severe hepatitis. Thus, halothane is not recommended in patients who have a family history of halothane sensitization [63][64][65]. In some studies, Caucasian background, obesity, age above 40-50 years and female gender are considered risk factors for the severe form of hepatitis [15][66][67].

## Enflurane, Sevoflurane, and Desflurane

Since the introduction of halothane, several halogenated anesthetics such as enflurane, desflurane, and sevoflurane have been developed. Post-exposure liver damage has been attributed to enflurane [50]. Based on the protein products of enflurane metabolism, it appears that cross-sensitization between halothane and enflurane is the likely cause of liver damage [45]. Nonetheless, the principal mechanism of liver damage following enflurane exposure is unknown [49][68]. Approximately 2% of enflurane undergoes hepatic metabolism compared with 30% of halothane [27]. Because hepatic metabolism, release of free radicals and the subsequent neo-antigen production constitute the likely mechanism of halogenated anesthetic-induced hepatotoxicity, enflurane appears to be less hepatotoxic compared with halothane. sevoflurane, another member of the family, is rapidly metabolized, releasing fluoride into the serum [69]. Although Sevoflurane is associated with liver metabolism (3% to 5%), it does not affect TFA production, the likely cause of neo-antigen formation [27]. Sevoflurane is not associated with elevated transaminase levels [70], but it decreases liver blood supply slightly in animals. Although this finding has not been demonstrated in humans, rendered it a safer compound compared with halothane [71]. Unlike sevoflurane, desflurane is metabolized in the liver to TFA. This may induce protein modification [72] and immune responses, especially in patients who are sensitized to halothane, but the metabolism rate is low (approximately 0.01%) [27]. Hence, the risk of fatal post-desflurane hepatitis is low compared with other halogenated anesthetics. However, few cases of hepatitis following desflurane might have been sensitized to halothane prior to desflurane exposure [47]. The true expense of implementing new anesthetics and substituting halothane is not well documented. These costs include the replacement of old anesthetic tools with newer methods of vaporizing agents and the higher price of new anesthetics. The costs of training medical staff should also be included. Further, the number of patients who need to be managed with new agents to avoid one case of halothane-induced adverse effects must be determined, as should the cost of managing that adverse effect [14]. Such estimates should be assumed in developing countries that have low health care budgets and can hardly afford the high costs of substituting old technologies with newer and safer ones. Nevertheless, the rare incidence of halothane-induced hepatitis in patients without any risk factors complicates the decision. A precise cost-benefit analysis must be performed in these countries before further action. In the absence of large-scale national studies, expert panels might be invaluable in determining the incidence of side effects.

## Discussion

Halothane can cause lethal hepatitis in patients who undergo general anesthesia. Thus, the most effective means of protecting patients is to avoid exposure. However, considering the high costs of implementing other drugs, especially in developing countries, and thfe rare occurrence of lethal diseases, halothane is the logical first option. Nonetheless, under special circumstances, as illustrated in Table 1 [19], when the probability of postexposure hepatitis is considerable, other anesthetics such as desflurane should be used.

**Table 1:** Contraindications for use of halothane, as defined by the Iranian Association of Gastroenterology and Hepatology

**Contraindications **(halothane or other inhalational anesthetics are contraindicated )	Halothane postexposure reaction history
	Recurrent exposure history
	Positive family history of postexposure reaction or halothane hepatitis
**High-risk patients** (halothane is not recommended in patients with 2 or more of these risk factors)	Female gender
	Age 40 years or more
	Obesity (BMI ≥ 28)
	Positive history of liver enzymes, inducing drugs, such as phenobarbital
